# Preparation and Characterization of a Novel *Longzhua mushroom* Polysaccharide Hydrogel and Slow-Release Behavior of Encapsulated Rambutan Peel Polyphenols

**DOI:** 10.3390/foods13111711

**Published:** 2024-05-29

**Authors:** Lingxin Zhao, Jiapeng Li, Yangyue Ding, Liping Sun

**Affiliations:** Faculty of Food Science and Engineering, Kunming University of Science and Technology, Kunming 650500, China; zhao13001662637@163.com (L.Z.); ljp134518@163.com (J.L.); dingyangyue77@163.com (Y.D.)

**Keywords:** *Longzhua mushroom*, mushroom polysaccharide, hydrogel, slow-release, rambutan peel polyphenols

## Abstract

Natural polyphenols have drawbacks such as instability and low bioavailability, which can be overcome by encapsulated slow-release systems. Natural polymer hydrogels are ideal materials for slow-release systems because of their high biocompatibility. In this study, Longzhua mushroom polysaccharide hydrogel (LMPH) was used to encapsulate rambutan peel polyphenols (RPP) and delay their release time to improve their stability and bioavailability. The mechanical properties, rheology, stability, swelling properties, water-holding capacity, RPP loading, and slow-release behavior of LMPH were investigated. The results showed that LMPH has adequate mechanical and rheological properties, high thermal stability, excellent swelling and water-holding capacity, and good self-healing behavior. Increasing the polysaccharide content not only improved the hardness (0.17–1.13 N) and water-holding capacity of LMPH (90.84–99.32%) but also enhanced the encapsulation efficiency of RPP (93.13–99.94%). The dense network structure slowed down the release of RPP. In particular, LMPH5 released only 61.58% at 48 h. Thus, a stable encapsulated slow-release system was fabricated using a simple method based on the properties of LMPH. The developed material has great potential for the sustained release and delivery of biologically active substances.

## 1. Introduction

Rambutan (*Nephelium lappaceum* L.) is an important tropical commercial fruit that can be eaten fresh or processed. The peel, rich in phenolic compounds, is discarded as a by-product during processing [1,2,3]. Rambutan peel polyphenols have high antioxidant and anti-inflammatory activities [4] but are susceptible to degradation, instability, and low bioavailability [5,6,7,8]. Encapsulation delivery systems have been used to improve the chemical stability of natural bioactives (e.g., polyphenols), particularly through the application of hydrogel systems [9].

Hydrogel is a substance with a three-dimensional network structure [10], capable of absorbing and holding large amounts of water, and has adjustable mechanical and chemical properties [11]. In particular, because of their porous structure, hydrogels are employed in the food industry as ideal materials for the encapsulation and delivery of bioactive substances [12,13]. At present, many researchers prefer to use natural polymers to prepare hydrogels [14]. The biocompatibility, accessibility, and renewability of natural polymers (e.g., polysaccharides) have led to their increased appeal as biomaterials compared to synthetic polymers [15].

Polysaccharides are macromolecular natural polymers. The hydroxyl and carboxyl groups on the polysaccharide molecular chain give the hydrogel excellent water retention properties [16,17]. In addition, natural polysaccharides can bind polyphenols through hydrogen bonding and hydrophobic interactions and immobilize them in the porous network of the hydrogel for slow release [18,19].

Natural polysaccharides are derived from various sources, including animals, plants, microorganisms, and algae [20]. These polysaccharides, including chitosan, starch, cellulose, and lignin, have been extensively used to prepare natural hydrogels [21]. In recent years, fungal polysaccharide resources have gained increased attention because of their rich availability and safety [22]. Mushroom is a higher edible fungus [23]. Mushroom polysaccharide is a non-animal source, so the risk of disease transmission is limited [24]. Moreover, mushroom polysaccharides have high bioactivity and gelling properties and, thus, have promising applications in tissue engineering and encapsulation [25,26]. Most studies have described mushroom polysaccharides mixed with other substances to construct gel systems. For example, polysaccharides isolated from *Agaricus blazei* improved the gelling ability of Pluronic^®^ F127 systems [27]. In the study by Zhang et al. [24], *Pleurotus tuber-regium* was compounded with xanthan gum to construct a hydrogel for encapsulation. The bio-hydrogels prepared by mixing the polysaccharides of *Hericium erinaceus* and hydrolyzed extracts of *Paulownia elongata x fortunei* showed high stability [28]. In addition, some studies on single-component polysaccharide hydrogels have mostly been conducted by changing the pH and adding ions to make them gelatinous; for example, *Poria cocos* polysaccharides were acidified to make them gelatinous by Li et al. [20]. These methods have the potential to affect the structural and functional activity of the encapsulated substance. Therefore, there is an urgent need for a natural polysaccharide that can autonomously form weak gels to construct hydrogel systems.

At present, reports on the study and application of this single-component polysaccharide and its gelling properties are rare. Longzhua mushroom is a new variety obtained by controlling the growth and developmental conditions of *Auricularia polytricha* to make it look like a claw. Longzhua mushroom has the same rich polysaccharide content as *Auricularia polytricha*, and we hypothesize that its polysaccharides have gelling properties similar to those of *Auricularia polytricha*.

The aim of this study was to construct a simple, green, safe and stable single-component hydrogel as an encapsulation delivery system, using Longzhua mushroom polysaccharide (LMP) to slow down the release rate of rambutan peel polyphenols (RPP). The mechanical properties, microstructure, self-healing ability, stability, water-holding capacity, and swelling properties of the hydrogel were investigated. We hope that this study will provide a novel, safe, and easily accessible material for single-component hydrogels and expand the research related to LMP.

## 2. Materials and Methods

### 2.1. Materials

Fresh Longzhua mushroom was purchased from the wild mushroom market in Kunming, Yunnan Province, China. Fresh rambutan was purchased from the fruit market in Kunming. Anhydrous ethanol was obtained from Zhiyuan Co., Ltd. (Tianjin, China). Folin phenol reagent was purchased from Macklin Biochemical Science and Technology Co., Ltd. (Shanghai, China). Methylene blue was obtained from Aladdin Biochemical Technology Co. (Shanghai, China). FT-IR grade KBr was purchased from Aladdin Reagent (Shanghai, China). Na_2_CO_3_ was purchased from Macklin Biochemical Science and Technology Co., Ltd. (Shanghai, China). All reagents were of analytical grade.

### 2.2. Preparation of Longzhua mushroom Polysaccharide (LMP) Hydrogel

The LMP extraction process was based on the method of Sun et al. [29]. Longzhua mushroom powder was soaked in 95% ethanol (1:15, *w*/*v*), then ultrasonicated twice for 30 min each time. The mixtures were centrifuged at 5000 rpm for 10 min, and the sediment was retained. Ultra-pure water (900 mL) was added to the sediment and extracted at a constant speed of 1000 rpm at 95 °C for 3 h. The mixture was then centrifuged at 5000 rpm for 15 min, and the supernatant was collected and concentrated under reduced pressure. The solution was mixed with four volumes of anhydrous ethanol and precipitated for 12 h. The precipitates were collected and lyophilized to obtain LMP.

LMP (100, 200, 300, 400, and 500 mg) and 10 mL of ultra-pure water were weighed and heated at 80 °C for 10 min while stirring and then stored at 4 °C. The hydrogels were named LMPH1–LMPH5, with the polysaccharide content ranging from 1% to 5% (*w*/*v*).

### 2.3. Texture Properties of LMPH

The hardness, springiness, cohesiveness, chewiness, and resilience of the hydrogel samples were measured using a texture analyzer (TA-XT PlusC, Stable Micro Systems, Godalming, UK) in TPA mode according to a previous method, with some modifications [30]. A P/36R probe was used for compression. The pre-test, test, and post-test speed was 1 mm/s. The determination time interval was 1.00 s, the compression distance was 5 mm, and the thixotropic force was 1.0 g. All measurements were conducted a minimum of five times.

### 2.4. Rheological Properties of LMPH

Rheological analyses were performed with a modular advanced rheometer (MCR 102, Anton Par, Graz, Austria) [31]. A 25-mm-diameter pressure plate (PP25) was used as the suspension measurement system at 25 °C, and the measurement gap was set to 1 mm.

#### 2.4.1. Viscosity Sweep

A shear rate of 0.1 to 100 s^−1^ at 25.0 °C was used to determine the apparent viscosity of all samples, and a total of 41 data points were collected.

#### 2.4.2. Strain Sweep

The linear viscoelastic range (LVR) was determined by adjusting the strain in the range of 0.01–1000% at a 1 Hz frequency. A total of 30 data points were collected. To avoid breaking the gels, subsequent rheological tests were performed within 1% of the strain.

#### 2.4.3. Frequency Sweep

Frequency sweep tests ranged from 0.1 to 100 rad/s. A total of 21 data points were collected. The results of the elastic (G’) and viscous (G”) moduli were recorded.

### 2.5. Scanning Electron Microscope (SEM)

The internal structure of LMPH was observed using a scanning electron microscope (Helios 5 CX, Thermo Scientific, Waltham, MA, USA). Before observation, the samples were lyophilized using a freeze dryer, then cut into small pieces with a sterilized scalpel, and spluttered with gold [32]. Images were observed and recorded at different magnifications.

### 2.6. Self-Restoring Characteristic

#### 2.6.1. Macro Self-Recovery Performance

Macro self-recoveries refer to the Wei et al. [33] method and make some modifications. Two hydrogels of different colors were prepared, one stained with methylene blue and the other left untreated. The hydrogels were left at room temperature and observed for self-healing without any external stimuli, and after 6 h, the hydrogels were lifted with forceps and recorded with a camera.

#### 2.6.2. Micro Self-Recovery Performance

The continuous step-strain test was performed at 1 Hz in the LVR (1% strain) and out of the LVR (1000% strain). The hydrogel was held for 150 s in each scanning segment, and the test was repeated five times at 25.0 °C [31].

The FTIR spectra of the samples were obtained by the KBr press method on a NICOLET iS50 Fourier transform infrared spectrometer (Thermo Scientific, USA). All spectra were recorded from 4000 to 400 cm^−1^ with 64 scans and a 1 cm^−1^ resolution.

### 2.7. Stability of LMPH

#### 2.7.1. Differential Scanning Calorimetry (DSC)

The thermal properties of LMPH were measured using a differential scanning calorimeter (DSC214, NETZSCH, Selb, Germany). LMPH (10 mg) was weighed into an aluminum pan, and an empty aluminum pan was used as a reference [34]. The temperature was increased from 20 °C to 200 °C at a rate of 10 °C/min, with a nitrogen flow rate of 40 mL/min [35].

#### 2.7.2. Freeze–Thaw Stability

The hydrogel was heated at 80 °C for 15 min to prepare a solution and expanded at 25 °C overnight. An empty tube was weighed. The solution was transferred into the empty tube. The tubes and solution were weighed and stored at −20 °C for 20 h, then thawed at 25 °C for 4 h as a freeze–thaw cycle. The entire experiment comprised five freeze–thaw cycles. At the end of each thawing cycle, LMPH was centrifuged at 5000 rpm for 10 min, and the separated water was weighed. Equation (1) was used to calculate the water separation rate.

(1) $$\mathrm{Water}\;\mathrm{separation}\;\mathrm{rate}=\frac{M_{2}}{(M_{1}-M)}\;\times \;100\%$$

where M is the weight of the empty tube, M_1_ is the total weight of the sample and centrifuge tube, and M_2_ is the weight of water separated after centrifugation.

### 2.8. Water Holding Capacity and Swelling Properties of LMPH

#### 2.8.1. Swelling Property

The swelling behavior of LMPH was investigated by gravimetric analysis. Hydrogels with different mass fractions were prepared in a 5 mL centrifugal tube. After vacuum freeze-drying, the dried LMPH was weighed 100 mg and placed on a 200-mesh filter. The LMPH was then immersed in ultrapure water and removed at 0.5, 1, 2, 3, 6, 9, 12, 24, 36, and 48 h, and any water present on the surface was quickly dried and then weighed. The expansion rate was calculated using Equation (2):

(2) $$\mathrm{Swelling}\;\mathrm{ratio}=\frac{\left(W_{1}-W_{0}\right)}{W_{0}}\;\times \;100\%$$

where W_1_ and W_0_ are the weight of the swollen hydrogel at the predetermined time interval and the initial weight of the lyophilized hydrogel, respectively.

#### 2.8.2. Water Holding Capacity

LMPH (20 mL) was prepared in a 50 mL centrifuge tube and then placed at room temperature for 2 h. The hydrogel was then centrifuged at 5000 rpm for 15 min. The water layer was removed from the gel surface with a dry filter paper until a constant weight was obtained. The WHC was calculated using Equation (3):

(3) $$\mathrm{WHC}=\frac{\left(M_{2}-M_{0}\right)}{\left(M_{1}-M_{0}\right)}\;\times \;100\%$$

where M_2_, M_1_, and M_0_ are respectively the weight of the centrifuge tube containing hydrogel after water removal, the initial weight of the centrifuge tube containing hydrogel, and the weight of the blank centrifuge tube.

### 2.9. In Vitro Sustained Release Behavior

#### 2.9.1. Encapsulation Efficiency (EE)

Rambutan peel polyphenols (RPP) powder from our previous work [36]. RPP suspension was prepared by taking 10 mg of RPP dispersed in 10 mL of ultrapure water. LMP powder (100, 200, 300, 400, 500 mg) was added to 10 mL of RPP suspension and stirred at 80 °C for 15 min to completely dissolve the LMP powder to form five hydrogel samples of embedded RPP. The hydrogel was washed with 20 mL of ultrapure water, and 0.5 mL of the eluate was added with 2.5 mL of 10% Folin phenol, vortexed, and left to stand for 5 min, followed by the addition of 2 mL of Na_2_CO_3_, and the reaction was carried out at the dark place for 1 h. The polyphenol content of the washings was determined at 765 nm [37]. The encapsulation efficiency of rambutan polyphenol was determined using Equation (4):

(4) $$\mathrm{EE}=\left(1-\frac{A_{2}}{A_{1}}\right)\;\times \;100\%$$

where A_1_ and A_2_ are absorbance values of RPP solution and cleaning solution, respectively.

#### 2.9.2. In Vitro Release Behavior

The freeze-dried hydrogel containing RPP was immersed in 50 mL of deionized water at 37 °C, and the oscillation was maintained at 100 rpm during the experiment. The solution (2 mL) was removed at 0.5, 1, 2, 3, 6, 9, 12, 24, 36, and 48 h, and replaced with the same volume of deionized water to maintain a constant volume [38]. The collected samples were analyzed based on the standard curve to determine the released amount of RPP. The cumulative release of RPP was calculated using Equation (5):

(5) $$\mathrm{Cumulative}\;\mathrm{Release}=\frac{C_{t}V_{0}+\sum_{1}^{t-1}V_{s}C_{t}}{m}\;\times \;100\%$$

where C_t_ is the concentration of RPP in the solution at the time of sampling (mg/L); V_s_ is the volume of each sample (L); V_0_ is the total volume of the solution (L); m is the drug-carrying capacity of the hydrogel (mg).

### 2.10. Statistical Analysis

All analyses were performed in triplicate. Means, standard deviations, and graphs were obtained using Origin 2023. Data sets were evaluated using one-way ANOVA (Duncan test) with a significance of *p* < 0.05. Statistical analyses were performed using the SPSS 21 package for Windows.

## 3. Results and Discussions

### 3.1. Texture Property

Texture is an important index to evaluate the structural stability of hydrogels. The texture measurements of LMPH are shown in Table 1. The hardness of LMPH increased substantially from 0.17 N (LMPH1) to 1.13 N (LMPH5). As a result of the increase in the content of Longzhua mushroom polysaccharide, crosslinking increased, which caused the hydrogel to form numerous hydrogen bonds and a dense network structure, resulting in an increase in hardness [32,39]. Generally, the structural integrity of a hydrogel is directly proportional to its hardness; the higher the hardness of a hydrogel, the higher its structural integrity [40]. The structural integrity of LMPH5 was significantly higher than that of LMPH1.

Chewiness can be a comprehensive response to the work performed by the chewing process of the sample, chewiness is positively correlated with hardness, the greater the hardness, the greater the work performed by chewing, so chewiness increases with increasing hardness (from 8.59 N to 82.55 N), which also reflects the structural stability of the LMPH is proportional to the polysaccharide content.

Cohesiveness is used to measure the internal bonding strength of the final structure of a hydrogel [41]. The cohesiveness of LMPH gradually increased, indicating an improvement in the internal hydrogel bond strength, which allowed the sample to withstand secondary deformation. LMPH5 exhibited the strongest cohesion (1.13). This indicates that an increase in the polysaccharide content promotes greater entanglement and the formation of stronger networks, leading to greater resistance to deformation [42]. The hydrogels prepared by Li et al. [43] had a maximum hardness of up to 0.9 N, a maximum chewiness of up to 3 N, and a maximum cohesiveness of up to 0.8, compared to which the hardness, chewiness, and cohesiveness of LMPH were significantly higher, which demonstrates that LMPH possesses better textural properties.

Springiness is used to describe the ability of hydrogels to bounce back. In general, an increase in hardness and cohesiveness leads to an increase in plasticity and rigidity, consequently resulting in reduced springiness of the hydrogels. However, the springiness of LMPH did not decrease significantly with increasing hardness. This result suggests that polysaccharide molecules form more hydrogen bonds within the hydrogel, resulting in a stronger hydrogel structure [44]. Resilience indicates the ability of a sample to bounce back during the first compression process. The recovery of LMPH5 decreased substantially compared with that of LMPH4 (0.35 to 0.27). Because of excessive crosslinking inside the hydrogel, the strong intermolecular interactions limit the movement of the molecular chains, resulting in longer sample recovery times. There have been cases where excessive crosslinking has reduced mechanical properties in similar experiments [45].

### 3.2. Rheology Analysis

**Figure 1:**
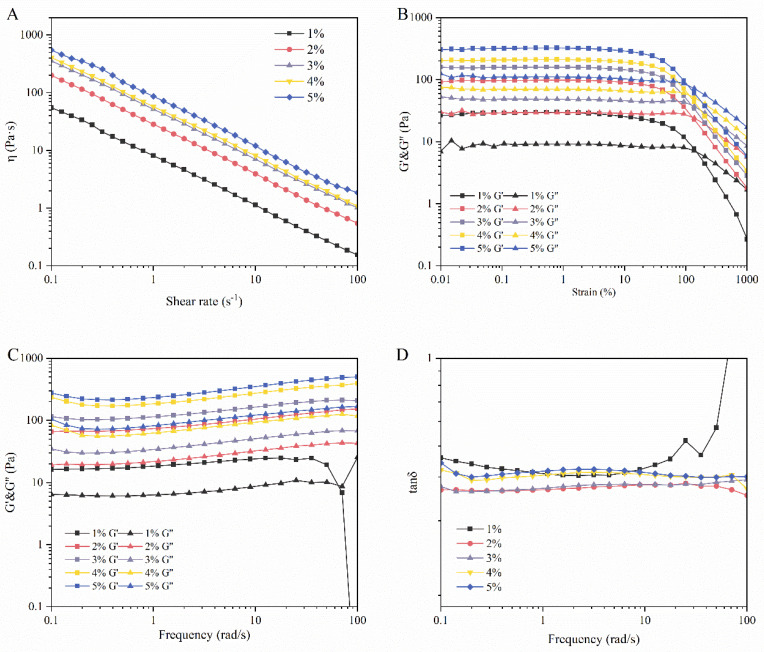
Rheological properties of LMPH. (**A**) Viscosity sweep; (**B**) LVR; (**C**) frequency sweep; (**D**) loss factor.

Rheological properties are crucial for the stability and viscoelastic behavior of hydrogels. The viscosity sweep of the LMPH is shown in Figure 1A. All gels showed substantial shear thinning. This shear-dependent behavior can be explained by the disruption of the low-energy intermolecular bonds between the polysaccharide chains, which is typical of weak gels [46]. This feature is common in plant polysaccharides, such as *Tremella* polysaccharide gel and *Dendrobium officinale* polysaccharide gel [47,48], which are pseudoplastic fluids. All pseudoplastic hydrogels exhibit shear-thinning properties. Shear-thinning systems have been widely utilized in tissue engineering and the delivery of bioactive substances [49]. In addition, the viscosity of samples increased as the proportion of the polysaccharide increased (i.e., LMPH5 was the most viscous), which suggests that LMPH forms a dense network structure. The increase in hydrogen bonding with increasing polysaccharide concentration leads to the tight connection between the polysaccharide molecules, restricting their movement and thus increasing the viscosity of LMPH [50,51,52].

As shown in Figure 1B, G’ was constant in the range of 0.1–10%. To obtain more information about the gel structure and mechanical properties, frequency sweep tests were performed within the LVR (1%). Figure 1C shows that LMPH2–LMPH5 exhibited G’ > G” in the frequency range of 0.1–100 rad/s, suggesting the presence of an interconnected network structure and indicating excellent stability against mechanical stress [53]. However, when the angular frequency increased, LMPH1 behaved as G” > G’, and the hydrogel exhibited a viscous behavior. This result is similar to that of tanδ (Figure 1D). As the angular frequency increases, LMPH1 conforms to tanδ > 1, and the LMPH1 system exhibits the characteristics of liquid viscosity. This phenomenon is related to the slippage effect caused by the small-layer synergistic effect of partial sample decomposition [54,55]. In addition, the G’ of LMPH5 was the highest, which means that when the hydrogel resisted deformation, most of the deformation energy was stored in the LMPH5. Moreover, the network structure of this hydrogel is denser, resulting in increased deformation resistance, which is consistent with the texture measurement. The results in Figure 1D are similar to those in Figure 1C, LMPH2–LMPH5 as tanδ < 1, the system mainly exhibits solid elastic properties [56].

### 3.3. The Self-Healing Ability of LMPH

#### 3.3.1. Macroscopic Morphology of LMPH Self-Healing Process

**Figure 2:**
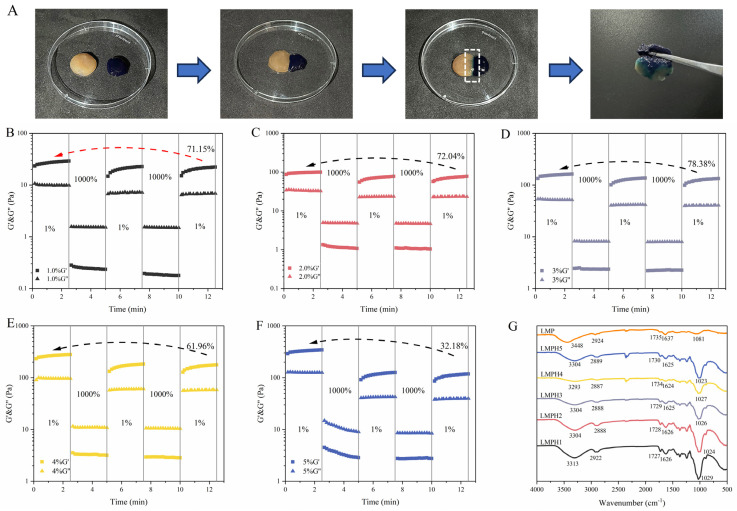
Self-healing properties of LMPH. (**A**) Macroscopic self-healing behavior of cut hydrogels at room temperature; (**B**–**F**) continuous step-strain tests of the hydrogel; (**G**) FT-IR spectroscopy of LMP and LMPH.

The macroscopic self-healing properties of LMPH are shown in Figure 2A. The methylene blue-treated hydrogel and the untreated hydrogel were cut into two pieces and spliced together. After 2 h at room temperature, the boundary between the two hydrogels became blurred. After 6 h, the hydrogel did not break when picked up with tweezers. This suggests that the hydrogen bonding between the polysaccharide molecules contributes to the self-healing properties of the hydrogel rather than simple adhesion occurring at the interface of hydrogel fragments [57].

#### 3.3.2. Time Sweep

The self-healing ability of LMPH was tested by changing the shear strain. As shown in Figure 2B–F, the rheological behavior of LMPH within the LVR (1% strain) was elastic (G’ > G”), whereas, at larger strain scans (1000% strain), LMPH shifted to a viscous behavior (G’ < G”). When the shear stress returned to 1%, G’ recovered and the hydrogel showed elastic properties again, suggesting recovery of the gel network and demonstrating the good self-healing ability of LMPH [58]. However, the hydrogel could not reach the original G’ value. The self-healing abilities of LMPH1–LMPH5 were different. LMPH3 exhibited the best self-healing performance, and a 78.38% recovery was reached after two consecutive structural failures. LMPH5 showed the worst recovery (only 32.18%) compared to the Poria polysaccharide hydrogel prepared by Li et al. (minimum 62.25%) [20]. This is because its higher polysaccharide concentration promotes higher entanglement and stronger network formation; thus, it limits chain motion and requires a longer time to bring the system back to equilibrium [42].

#### 3.3.3. FT-IR

The FT-IR spectra of LMP and LMPH are shown in Figure 2G. The peaks at 3448, 2924, 1735, 1637, and 1081 cm^−1^ were attributed to the O-H stretching vibration, C-H stretching vibration, COO- stretching vibration, and alcohol hydroxyl variable angle vibration of LMP [59]. Compared with LMP, the characteristic peaks in the spectrum of LMPH all appear at positions with a low vibration frequency. The peaks of LMP at 3448, 1735, and 1081 cm^−1^ are shifted to positions around 3304, 1730, and 1027 cm^−1^ in LMPH.

The positions of these peaks are redshifted, indicating the presence of hydrogen-bonding interaction forces within the hydrogel [60,61,62]. The formation of hydrogen bonds reduces the density of the bonding electron cloud, and stretching vibration absorption shifts to a low wavenumber [63]. LMPH is mainly formed by hydrogen-bonding crosslinking. As a result of hydrogen-bonding interactions and complexation between the polysaccharide molecules in the network, the polymer chains on the surface of the hydrogel wounds are constantly moving and re-crosslinking, thus restoring the hydrogel network at the molecular level [64,65]. Thus, we hypothesize that the self-healing ability of hydrogels is caused by the presence of intermolecular hydrogen bonding and complexation [66].

### 3.4. SEM

**Figure 3:**
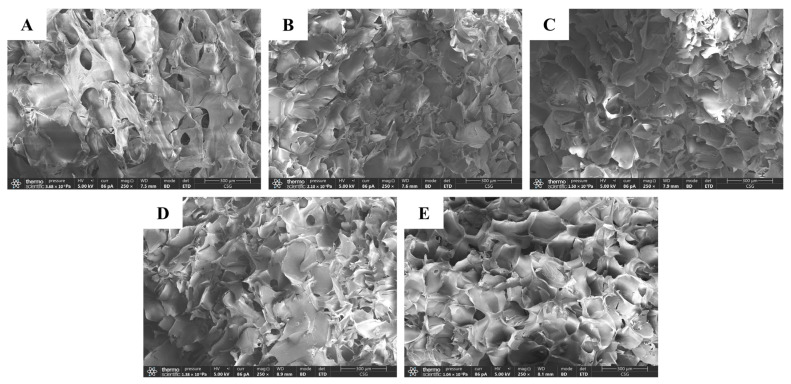
SEM images of LMPH. (**A**) LMPH1; (**B**) LMPH2; (**C**) LMPH3; (**D**) LMPH4; (**E**) LMPH5.

Considering the slow-release application of hydrogels in this study, knowledge of their microstructure can provide information on the synergistic relationship between their swelling behavior and release behavior [67]. Figure 3 shows the network structure of LMPH. The hydrogel exhibits a porous structure, and the pore size is in the range of 50–200 μm. The porous structure of LMPH can provide a larger surface area and pore capacity, which is conducive to the adsorption, storage, and release of substances, making LMPH ideal for loading and embedding bioactive substances for slow release [68,69]. From LMPH1 to LMPH5, the network structure gradually becomes more organized and denser. Nevertheless, if the network structure of the hydrogel is too ordered and dense, it will limit the penetration and diffusion of water molecules, reduce swelling, and slow down the release rate of bioactive substances [70].

### 3.5. Water Hold Capacity and Swelling Rate

#### 3.5.1. WHC

**Figure 4:**
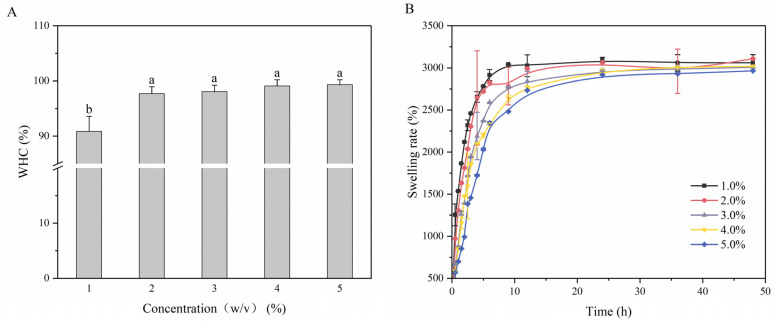
Water retention and swelling properties of LMPH. (**A**) WHC; (**B**) swelling rate. Different lower-case letters represent significant differences between samples.

The WHC of a hydrogel represents its ability to retain water under external forces [71,72]. As shown in Figure 4A, the lowest WHC of LMPH was 90.84%, and the highest WHC of the hydrogel prepared by He et al. [71] was only 75.57%, indicating that the water-holding property of LMPH was superior. With the increase of polysaccharide content, the WHC also increased, and the WHC of LMPH5 increased significantly to 99.32%.

The number and density of gel networks are two key factors affecting WHC. The WHC of LMPH1 is low because of the loose gel networks, whereas LMPH5 has the highest WHC because it has the highest polysaccharide content, contains more hydroxyl groups, and has high hydrophilicity [73]. A high polysaccharide content results in the formation of a dense network structure of hydrogen bonds through interactions, and a large amount of water is immobilized by physical interception [74].

#### 3.5.2. Swelling Rate

Swelling capacity is another key property of hydrogels as it reflects their ability to adsorb large amounts of water and is reflective of their internal structure [75]. As shown in Figure 4B, LMPH reached swelling equilibrium within 15 h, and the swelling ratio exceeded 2500%. As the polysaccharide content increased, the swelling rate decreased from 3129.94% (LMPH1) to 2965.88% (LMPH5). The hydrogen bonding interactions between –OH groups in the polysaccharide molecules prevent water from entering the interior of the hydrogel, resulting in a low swelling ratio [76]. This outcome was consistent with Y. Wang et al. [70], who reported that a dense crosslink density hindered the diffusion of water, as demonstrated by the water-holding capacity test.

### 3.6. Stability

#### 3.6.1. Differential Scanning Calorimetry (DSC)

**Figure 5:**
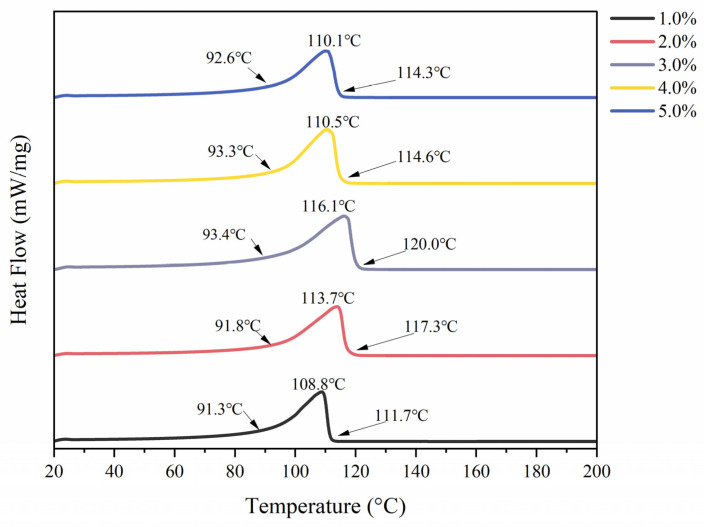
DSC curves of LMPH.

The thermal stability of the LMPH with different polysaccharide contents was analyzed by DSC. The LMPH exhibited an endothermic peak (Figure 5), indicating that melting and decomposition took place. The endothermic peak at approximately 110 °C is related to the thermal decomposition of polysaccharides, including the hydrogen bond between galacturonic acid units and the conformational change, decarboxylation of side-chain groups, and carbon in the ring [77]. The temperature at which LMPH1 and LMPH3 began to decompose increased from 91.3 °C to 93.4 °C, and the peak temperature of decomposition increased from 108.8 °C to 116.1 °C, respectively. The higher temperature required for decomposition demonstrated that the increase in the content of the polysaccharide enhanced the thermal stability of LMPH [78]. The higher values imply higher stability caused by stronger bonds within the chains due to bond formation, loss of internal water, or other reasons [79,80]. In summary, LMPH shows high thermal stability, ensuring the stable and sustained release of bioactives at high temperatures.

#### 3.6.2. Freeze–Thaw Stability

The experimental results are summarized in Table 2. As the freezing and thawing cycles increased, the water separation rate of LMPH first increased and then decreased. The low water separation rate in Cycle 1 is due to the initial formation of the gel network of LMPH, which is less affected by the freeze–thaw cycle. However, as the number of freeze–thaw cycles increases, the water separation rate of LMPH increases, indicating that the freeze–thaw cycle causes great damage to the network structure of LMPH [81]. The formation and melting of ice crystals during freezing and thawing disrupt the network structure of the hydrogel, resulting in the disruption of the hydrogen bonds between the polysaccharide molecules [82]. For Cycle 3, the water separation rate of LMPH1 reached 34.65%. Then, the water separation rate started to decrease, and the water separation rate of LMPH1 was 12.25% in Cycle 5. The freeze–thaw stability of LMPH was significantly better compared with the prepared ginkgo polysaccharide hydrogels (with a minimum water separation rate of not less than 70%) of Zhang et al. [34]. This shows that the network structure of LMPH is gradually improved, the freeze–thaw stability of the hydrogel is gradually improved, and the infiltration and separation of water are reduced.

The higher the polysaccharide content in LMPH, the better its freeze–thaw stability. The freeze–thaw stability is related to viscosity [83]. LMPH5 exhibited the highest freeze–thaw stability because it had the highest viscosity. The hydrogen bonds in the polysaccharide molecules increase the viscosity of the gel and the strength and stability of the gel network. The high viscosity limits the separation of water [84,85]. In general, the denser the gel network, the stronger the water-binding capacity. This result is consistent with the rheology results (static viscosity sweep tests).

### 3.7. In Vitro Release Behavior

#### 3.7.1. Encapsulation Efficiency (EE)

**Figure 6:**
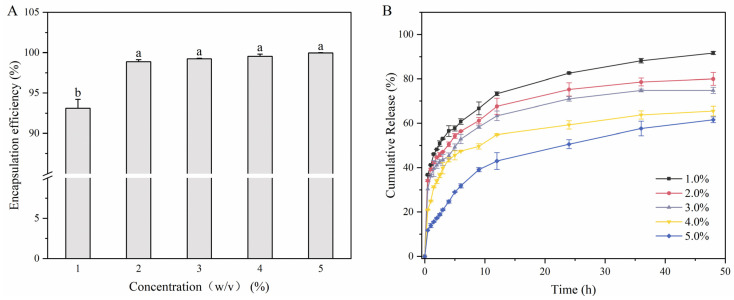
Encapsulation efficiency and release behavior of RPP in vitro. (**A**) Encapsulation efficiency of RPP; (**B**) in-vitro release behavior. Different lower-case letters represent significant differences between samples.

As shown in Figure 6A, the EEs were 93.13% (LMPH1), 98.89% (LMPH2), 99.24% (LMPH3), 99.56% (LMPH4), and 99.94% (LMPH5). EE is proportional to the polysaccharide content in LMPH. A high EE is related to the porous structure of the hydrogel, which adsorbs more bioactive molecules. As the polysaccharide content increases, the stronger the interactions of hydrogen bonds formed between polysaccharide molecules, the more ordered the network structure is formed, and the more ordered network structure can provide more contact and immobilization points for RPP. RPP was captured by the network structure of the hydrogel. When the hydrogel is rinsed with ultrapure water, it is difficult for water molecules to enter the hydrogel with high polysaccharide concentration and for RPP molecules to separate from the dense network structure. As a result, the polysaccharide concentration of the hydrogel is higher, and the encapsulation efficiency is higher. By tuning the porous structure and network organization of the gels, higher encapsulation efficiency can be achieved, expanding the potential of hydrogels for nutrient delivery applications.

#### 3.7.2. In Vitro Release Behavior of Polyphenols from Rambutan Peel Polyphenols

Based on the above-mentioned porous structure and swelling properties of LMPH, this study used LMPH as a slow-release material and rambutan peel polyphenol as a model bioactive substance. Figure 6B shows the sustained release of rambutan peel polyphenols by LMPH within 48 h. The rapid release trend of polyphenols during the first 5 h was attributed to the inability of the cross-linked network of the hydrogel to control surface drug release when the hydrogel was in a release medium that allowed free initial dissolution of polyphenols. With time, the release of polyphenols gradually increased. LMPH1 showed the highest cumulative polyphenol release rate (91.70%). LMPH5 had the lowest cumulative release rate (61.58%). Just as high-concentration polysaccharide hydrogels prevented water molecules from entering the gel interior in swelling experiments and hydrogels trapped water molecules in water retention experiments, the dense network structure limited interchain repulsion, slowed the penetration of water molecules into the interior of the hydrogel, and allowed for better sequestration of polyphenols [86]; a result consistent with those of M. Li et al. [43], where the release rate of substances inside the hydrogel was reduced due to a denser network structure inside the hydrogel, and the dense network structure negatively affected the release behavior.

## 4. Conclusions

In this study, a novel one-component hydrogel was successfully prepared by heating using polysaccharides from Longzhua mushroom. Compared with the common methods of preparing hydrogels, which usually require the addition of an inducer to make them gel or the compounding of two or more substances to form a gel, the preparation method in this study is simple and easy to use. Then, Longzhua mushroom polysaccharide hydrogel (LMPH) was used to study the encapsulation and slow release of rambutan peel polyphenols (RPP). The rheological and textural properties of the LMPH were characterized. All the hydrogels showed adequate viscoelasticity and textural properties. LMPH exhibited good self-healing properties driven by hydrogen bonding. LMPH has a three-dimensional porous network structure, and its water-holding capacity (WHC) and swelling rate are related to its porous structure. The WHC and swelling properties of the hydrogel were correlated with the polysaccharide content of LMPH, with WHC being directly proportional to the polysaccharide content and swelling rate being inversely proportional to the polysaccharide content. LMPH was evaluated for the slow release of RPP, which lasted for 48 h. These results suggest that the novel LMPH has adequate mechanical and self-healing properties as well as stability and thus has a wide range of potential applications in the slow release of nutrients and delivery of bioactive substances.

## Figures and Tables

**Table 1:** Textural property of LMPH.

Sample	Hardness (N)	Chewiness (N)	Cohesiveness	Springiness (mm)	Resilience
LMPH1	0.17 ± 0.02 ^d^	8.59 ± 0.62 ^d^	0.92 ± 0.06 ^b^	0.55 ± 0.04 ^a^	0.26 ± 0.04 ^b^
LMPH2	0.26 ± 0.01 ^d^	15.53 ± 1.89 ^d^	1.05 ± 0.06 ^a^	0.56 ± 0.06 ^a^	0.27 ± 0.02 ^b^
LMPH3	0.61 ± 0.05 ^c^	39.03 ± 6.28 ^c^	1.07 ± 0.04 ^a^	0.60 ± 0.11 ^a^	0.31 ± 0.03 ^ab^
LMPH4	0.90 ± 0.06 ^b^	62.29 ± 4.39 ^b^	1.09 ± 0.03 ^a^	0.63 ± 0.03 ^a^	0.35 ± 0.04 ^a^
LMPH5	1.13 ± 0.18 ^a^	82.55 ± 8.39 ^a^	1.13 ± 0.09 ^a^	0.66 ± 0.06 ^a^	0.27 ± 0.05 ^b^

Each value represents the mean ± SD (*n* = 5). Values with different letters in the same column differ significantly (*p* < 0.05).

**Table 2:** The water separation rate (%) of LMPH in each cycle.

Concentration	Water Separation Rate (%)				
	Cycle 1	Cycle 2	Cycle 3	Cycle 4	Cycle 5
LMPH1	1.26 ± 0.09	12.0 ± 1.89	34.65 ± 2.33 ^a^	21.73 ± 1.42 ^a^	12.25 ± 1.26
LMPH2	ND	1.61 ± 0.14	21.64 ± 2.18 ^b^	4.57 ± 0.72 ^b^	ND
LMPH3	ND	ND	10.97 ± 0.11 ^c^	1.21 ± 0.21 ^c^	ND
LMPH4	ND	ND	4.28 ± 0.12 ^d^	0.28 ± 0.03 ^c^	ND
LMPH5	ND	ND	0.57 ± 0.19 ^d^	ND	ND

Note: Different lowercase letters in the same column indicate significant difference (*p* < 0.05). ND: Not detected.

## Data Availability

The original contributions presented in the study are included in the article, further inquiries can be directed to the corresponding author.
